# Knockout and Double Knockout of Cathepsin K and Mmp9 reveals a novel function of Cathepsin K as a regulator of osteoclast gene expression and bone homeostasis

**DOI:** 10.7150/ijbs.72211

**Published:** 2022-08-29

**Authors:** Guochun Zhu, Wei Chen, Chen-Yi Tang, Abigail McVicar, Diep Edwards, Jinwen Wang, Matthew McConnell, Shuying Yang, Yang Li, Zhijie Chang, Yi-Ping Li

**Affiliations:** 1 State Key Laboratory of Membrane Biology, School of Medicine, Center for Synthetic and Systems Biology, Tsinghua University, 100084 Beijing, China; 2 Division in Cellular and Molecular Medicine, Department of Pathology and Laboratory Medicine, Tulane University School of Medicine, Tulane University, New Orleans, Louisiana, 70112, USA; 3 Department of Pathology, University of Alabama at Birmingham School of Medicine, Birmingham, Alabama 35294-2182, USA; 4 Department of Basic & Translational Sciences, School of Dental Medicine, University of Pennsylvania, Philadelphia, PA, USA

**Keywords:** Cathepsin K, MMP9, epigenetic regulators, osteoclast, bone resorption, histone modification, osteoporosis

## Abstract

Cathepsins play a role in regulation of cell function through their presence in the cell nucleus. However, the role of Cathepsin K (Ctsk) as an epigenetic regulator in osteoclasts remains unknown. Our data demonstrated that *Ctsk^-/-^Mmp9^-/-^* mice have a striking phenotype with a 5-fold increase in bone volume compared with WT. RNA-seq analysis of *Ctsk^-/-^*,* Mmp9^-/-^* and* Ctsk^-/-^/Mmp9^-/-^* osteoclasts revealed their distinct functions in gene expression regulation, including reduced *Cebpa* expression, increased *Nfatc1* expression, and in signaling pathways activity regulation. Western blots and qPCR data validated these changes. ATAC-seq profiling of *Ctsk^-/-^*,* Mmp9^-/-^,* and* Ctsk^-/-^/Mmp9^-/-^* osteoclasts indicated the changes resulted from reduced chromatin openness in the promoter region of *Cebpa* and increased chromatin openness in Nfatc1 promoter in *Ctsk^-/-^/Mmp9^-/-^* osteoclasts compared to that in osteoclasts of WT, *Ctsk^/-^* and *Mmp9^-/-^*. We found co-localization of Ctsk with c-Fos and cleavage of H3K27me3 in wild-type osteoclasts. Remarkably, cleavage of H3K27me3 was blocked in osteoclasts of *Ctsk^-/-^* and *Ctsk^-/-^/Mmp9^-/-^* mice, suggesting that Ctsk may epigenetically regulate distinctive groups of genes' expression by regulating proteolysis of H3K27me3. *Ctsk^-/-^/Mmp9^-/-^* double knockout dramatically protects against ovariectomy induced bone loss. We found that Ctsk may function as an essential epigenetic regulator in modulating levels of H3K27me3 in osteoclast activation and maintaining bone homeostasis. Our study revealed complementary and unique functions of Ctsk as epigenetic regulators for maintaining osteoclast activation and bone homeostasis by orchestrating multiple signaling pathways and targeting both Ctsk and Mmp9 is a novel therapeutic approach for osteolytic diseases such as osteoporosis.

## Introduction

Cell signaling and transcriptional regulation of osteoclast differentiation, activation, and lineage commitment have been studied, however epigenetic regulation of osteoclast differentiation, activation, and function remains poorly understood. The epigenetic modifications associated with an active chromatin state open the tightly packed chromatin to allow for binding of regulatory molecules, including transcription factors, to activate gene expression and conversely for the epigenetic modifications associated with repressive chromatin structure. Osteoclast (OC) function and bone resorption is a complex process requiring precise regulatory mechanisms, with incompletely understood epigenetic involvement 1. Enhanced OC differentiation and/or activity are major contributing factors in bone loss during various osteolytic bone diseases, including osteoporosis; while defective OC formation and/or activity results in bone defects such as osteopetrosis and pycnodysostosis. Importantly, osteoporotic fractures generate a heavy burden of morbidity and financial cost in the elderly population, with hip fractures leading to permanent physical disability, loss of self-sufficiency, and an increased risk of death 2.

We previously cloned and characterized *Ctsk* as a gene highly expressed in OCs and induced by RANKL during OC differentiation and activity 3, 4. *Ctsk*-deficient mice display an osteopetrotic phenotype with excessive trabeculation of the bone-marrow space and reduced osteoclast senescence 4. *Mmp9,* a member of matrix metalloprotease (MMP) family*,* is also highly expressed in OCs and plays a role in bone mineral matrix degradation during bone resorption 5-7. Mmp9 is critical in controlling angiogenesis during bone development which is, in turn, critical for the formation of endochondral bone 8-11. However, Ctsk deficiency does not completely block OC function and Mmp9 deficiency, by itself, does not result in a bone phenotype, which suggests potential complementary functions of Ctsk and Mmp9. Although the absence of a bone phenotype in *Mmp9^-/-^* mice could be due to compensatory functions of other Mmps.

Cathepsins and MMPs could play a role in regulation of cell differentiation and function through their presence in the cell nucleus and selective cleavage of key nuclear proteins or histone modifications 12-15. In addition, Mmp9 has been shown to be important to osteoclastogenesis gene expression through selective proteolysis of the histone H3 tail 14. Although osteoclast lineage commitment 16, differentiation 17, 18, and activation 19-21 have been studied at the transcriptional level, the epigenetic regulation of osteoclast terminal differentiation, activation, and function have not been thoroughly explored. H3K27me3 is one of the major epigenetic regulators of gene expression in cell differentiation and organism development 22, 23 and has been demonstrated to play an important role in regulation of osteoclastogenesis 24-26. However, the mechanism of how levels of H3K27me3 are regulated remains unclear. Cathepsins and MMPs have been reported to enter the nuclei of cells and function as proteolytic enzymes where they cleave important proteins involved in genome regulation, but the role of Ctsk, specifically, remains unclear. Interestingly, *Ctsk* is expressed in non-bone resorbing cells such as tendon-derived *Ctsk*-expressing progenitor cells 27 and *Ctsk*-expressing mesenchymal progenitor cells, in the groove of Ranvier 28. However, Ctsk's function in these cells does not seem to involve its canonical function in matrix protein degradation.

Considering potential complementary functions of Mmp9 and Ctsk as epigenetic regulators, we used *Ctsk*^-/-^, *Mmp9*^-/-^ and *Ctsk*^-/-^/*Mmp9*^-/-^ double knockout (DKO) mouse models to investigate the effects of cathepsin K in epigenetic regulation of osteoclast gene expression, and bone homeostasis more broadly. Our data demonstrated that *Ctsk^-/-^Mmp9^-/-^* mice have a striking phenotype with a 5-fold increase in bone volume compared with WT. Our data indicated that Ctsk plays a novel function as epigenetic regulators in osteoclast gene expression and activation. Ctsk may epigenetically modulate bone resorption and the expression of distinctive groups of genes in osteoclasts through regulating proteolysis of H3K27me3.

## Methods

### Animal Studies

WT, *Ctsk*-deficient (*Ctsk^+/-^* and *Ctsk^-/-^*), and *Mmp9*-deficient (*Mmp9^+/-^* and *Mmp9^-/-^*) mice were bred on the C57BL/6J background. *Ctsk*^-/-^/*Mmp9*^-/-^ DKO mice were generated by crossing *Ctsk*^-/-^ mice 4 with *Mmp9*^-/-^ mice 29. Both male and female mice of each strain were randomly selected and used with littermates where possible. The investigators were not blinded during allocation, animal handling, and endpoint measurements. The mouse procedures were approved by the University of Alabama at Birmingham Institutional Animal Care and Use Committee (IACUC) and the Tulane University IACUC, and followed the guidelines of the Animal Research: Reporting in Vivo Experiments (ARRIVE) 30.

### Ovariectomy

We performed surgical removal of the ovaries to induce osteoporosis. 2-month-old female mice were used in the ovariectomy experiments. We assessed the effects of ovariectomy (OVX) on bone mass in our DKO, and single knock out, 4-month-old mouse models. For ovariectomy, mice were anesthetized with isoflurane inhalant, one midline incision was made, and both gonads were exposed, ligated, and removed. Gut suture was used on the inner dermal layer and a single wound clip applied to suture the skin. After gonad removal, mice were administered a subcutaneous injection of 0.9% sodium chloride (for rehydration) and a topical analgesic (0.25% bupivacaine) and kept warm until awakening per Addison and Rissman 31. Sham surgery was also conducted in the same manner, without ligation and removal of the gonads. Successful removal of the ovaries was assessed visually at the endpoint based on uterine shrinkage.

### Radiological and Physical Skeletal Analysis

Skeletal phenotypes were determined histomorphometrically from the metaphyseal cancellous bone of distal femora. X-ray images were taken using a Faxitron MX-20 (Faxitron X-ray) by our collaborator Shuying Yang at the University of Pennsylvania. Micro-computed tomography (micro-CT/µCT) was performed using a Scanco Medical µCT 35 at the Small Animal Phenotyping Lab at the University of Alabama at Birmingham (UAB). Bone flexural testing was performed using the TA RSA-G2 three-point bend apparatus (TA Instruments, New Castle, DE, USA) in the UAB department of Biomedical Engineering.

### Bone Histology and Staining Analysis

TRAP (tartrate resistant acid phosphatase) stains for osteoclasts, ALP (alkaline phosphatase) stains for osteoblasts, and Von Kossa stain for bone mineralization were performed as previously described 16, 32, 33. H&E (hematoxylin and eosin) and trichrome staining (Goldner's Trichrome) were performed for assessing tissue and cellular morphology as previously described 16, 32, 33.

### Enzyme Linked Immunosorbent Assay (ELISA)

ELISA was performed according to manufacturer instructions. ELISA kits used were CTX/C-telopeptide assay for bone resorption (MyBioSource, cat# MBS724196), P1NP/total procollagen type 1 N-terminal propeptide (R&D Systems, cat# DY6220-05).

### Western Blotting

Western blotting was performed as previously described 33. Antibodies used are cathepsin k (Santa Cruz, cat# sc-48353), Mmp9 (Santa Cruz, cat# sc-6840), Gapdh (Santa Cruz, cat# sc-25778), phosphorylated Nfkb p65 (Cell Signaling, cat# 3033S), total Nfkb p65 (Cell Signaling, cat# 8242S), phosphorylated Akt (Cell Signaling, cat# 2965S), total Akt (Cell Signaling, cat# 2938S), H3 (Millipore/Sigma, cat#06-755), H3K36me3 (Millipore/Sigma, cat#05-801), H3K18ac(Millipore/Sigma, cat#07-354), H3K27me3 (Millipore/Sigma, cat#07-449), H3k4me1 (Millipore/Sigma, cat#07-436), H3k4me3 (Millipore/Sigma, cat#07-473), NFATC1 (Santa Cruz sc-7294), CEBPa (Santa Cruz sc-61), PU.1 (Santa Cruz sc-352),ATP6i (Santa Cruz sc-162300), c-Fos (Santa Cruz sc-52), Beta-Tubulin (E7; DSHB).

### *In vitro* OC Differentiation and Bone Resorption Assays

The osteoclastogenesis assays were carried as described in our previous studies 34-36. Assays include kits for TRAP (cat# 387A-1KT), acridine orange (cat# A-6014), and WGA-FITC (cat# L4895-2MG) or WGA-HRP (cat# L3892-1mg) staining (all from Millipore-Sigma; St. Louis, MO, USA) and actin ring staining with rhodamine-phalloidin (cat# R415; Thermo-Fisher; Waltham, MA, USA). Pit depth was assessed using confocal microscopy with a Nikon A1R confocal microscope in the UAB High Resolution Imaging Core Facility. A representative area for each assay was shown. Data were quantified by measuring the percent resorbed areas in three random areas using the ImageJ software from the National Institutes of Health.

### RNA-Sequencing Analysis

RNA-sequencing and analysis was performed as previously described 37. Total RNA was isolated using TRIzol reagent (Invitrogen Corp., Carlsbad, CA) from the osteoclasts that were cultured for 14 days in osteogenic differentiation medium following the manufacturer's protocol and was submitted to Admera Health (South Plainsfield, NJ) who assessed sample quality with the Agilent Bioanalyzer and prepared the library using the NEBnext Ultra RNA - Poly-A kit. Libraries were analyzed using Illumina next generation sequencing and relative quantification was provided by Admera Health. Read counts were subjected to paired differential expression analysis using the R package DESeq2. Volcano plot of differentially expressed genes was generated using log_2_ (fold change) and -log_10_ (p value) values. Genes were considered significant for upregulation/downregulation if *p* < 0.05. GO analysis was carried out using DAVID online tool (https://david.ncifcrf.gov/). Top GO downregulated categories were selected according to the *P*-values and enrichment score, and illustrated as number of genes downregulated in respective category. Signaling pathway data were analyzed by Ingenuity Pathway Analysis (IPA) (QIAGEN Inc., https://www.qiagenbioinformatics.com/products/ingenuitypathway-analysis). R to graph the differential expression genes analyzed by IPA.

### ATAC-seq

Mouse bone marrow cells were isolated from WT, Ctsk^-/-^, Mmp9^-/-^, and DKO (Ctsk^-/-^Mmp9^-/-^) mice and induced to osteoclastogenesis according to the lab's standard protocol 38. ATAC-seq chromatin samples were isolated using the protocol from Buenrostro et al. 39. Transposition and library preparation, using the Nextera library prep kit (Illumia, San Diego, CA), was performed along with HiSeq sequencing at Admera Healthcare (South Plainfield, NJ). Initial analysis including peak calling were also performed by Admera Health. Peaks were called using Macs2, first at sample level and then at group level by pooling samples together 40. GO analysis was performed in PANTHER 41. The peak plots were prepared using integrated genome viewer.

### Three-point bend assay

The RSA-G2 from TA Instruments (New Castle, DE) was used to perform a 3-point bend tensile strength test on mouse femurs from WT, *Ctsk^-/-^*, *Mmp9^-/-^*, and DKO (*Ctsk^-/-^*/*Mmp9^-/-^*) mice to determine their material characteristics including flexibility, and durability to strain by applying continuously increasing force and measuring deflection until the bones broke. Methods used are described in Bhuiyan et al 42 with the device in flow axial mode.

### Quantitative Reverse Transcription Polymerase Chain Reaction (qRT-PCR) analysis

The RNA extraction procedure was the same operation used in RNA-seq analysis. The total RNA was reverse transcribed into cDNA using RevertAid RT Kit (K1691, Thermo Scientific) according to the manufacturer's instructions. We carried out qPCRs by using SYBR Green Reagents (4309155, Thermo Scientific) and Step-One Real-Time PCR System (Life Technologies, Applied Biosystems) 20, 43. The expression of glyceraldehyde-3-phosphate dehydrogenase (*Gapdh*) was used as an endogenous control for normalization.

### Statistical Analysis

The number of animals used in this study was determined in accordance with our previous studies 44-46. In brief, the current study utilized 5 mice per group per experiment. Quantitative analysis was performed using GraphPad Prism. Experimental data are reported as averages ± SD of at least triplicate independent samples. Data were analyzed with two-tailed unpaired *t* test; P values <0.05 were considered significant. *P < 0.05; **P < 0.01; ***P<0.001; ****P<0.0001.

### Data Availability

The RNA-Seq data are available upon request from Yi-Ping Li, Department of Pathology and Laboratory Medicine, Tulane University School of Medicine, E-mail: yli81@tulane.edu. All other data are contained within the manuscript.

## Results

**Figure 1:**
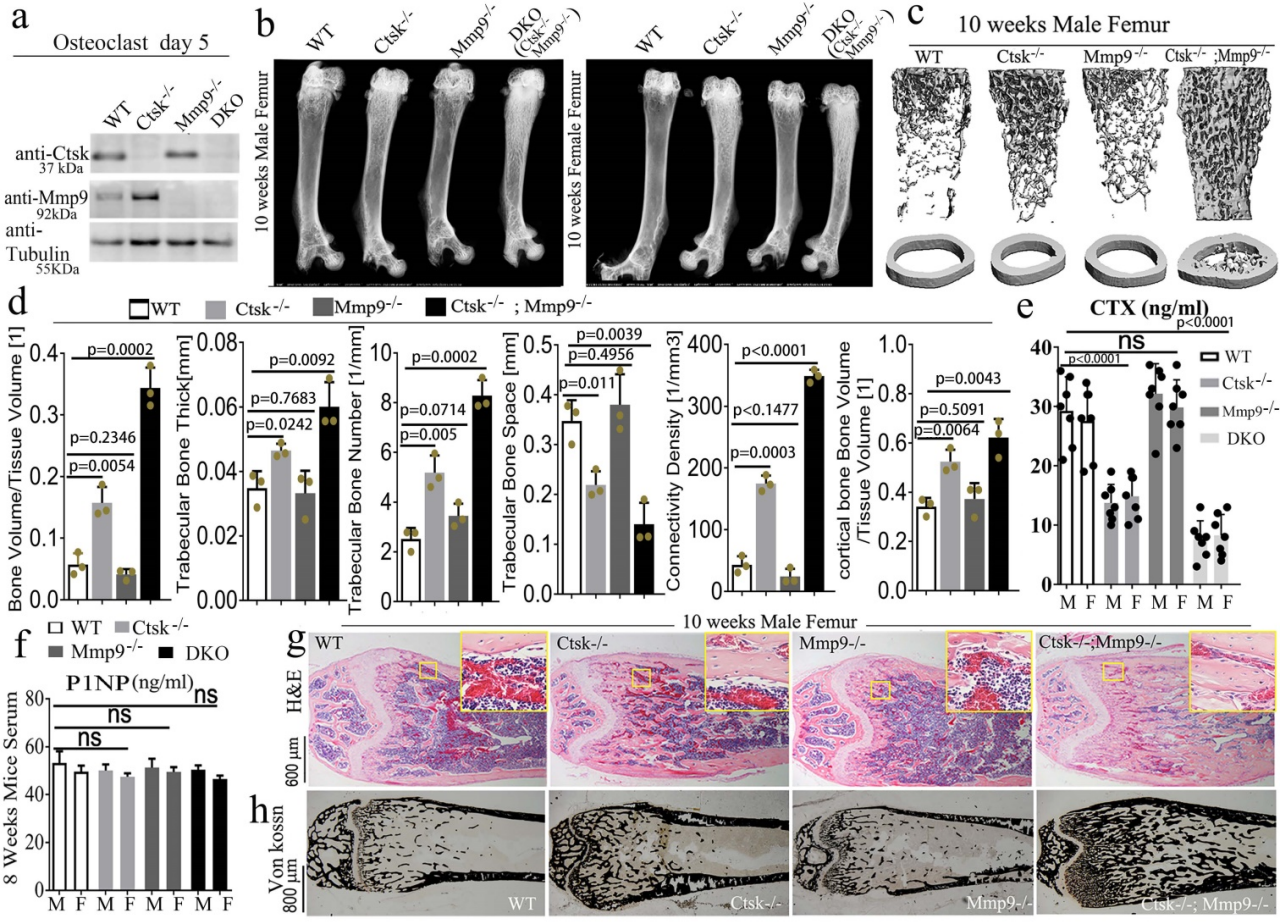
** Double deletion of *Mmp9* and *Ctsk* causes increased bone mass and severe osteopetrosis in mice. (a)** Western blotting validation of protein knockout in the mutant mice used with beta-Tubulin loading control (n=3 independent experiments). **(b)** X-ray analysis of 10-week-old mouse femurs, from male and female mice (n=10 animals). **(c)** MicroCT analysis of trabecular bone (n=3 independent experiments). **(d)** Quantification of microCT results in C. **(e)** Circulating c-telopeptide (CTX) levels from 8-week-old male (M) and female (F) mice (n=7 animals). **(f)** Circulating N-terminal type 1 collagen propeptide (P1NP) levels from 8-week-old mice (n=3 independent experiments). **(g)** Hematoxylin and eosin (H&E) staining of distal femurs from 10-week-old male mice (n=3 animals). **(h)** Von kossa staining of distal femurs from 10-week-old male mice (n=3 animals). All the data are presented as mean values ± SD; two-tailed unpaired *t* test; ns. non-significant.

**Double deletion of *Mmp9* and *Ctsk* causes increased bone mass and severe osteopetrosis in mice.** To determine whether *Ctsk* may compensate for the loss of *Mmp9* in *Mmp9*^-/-^ mice during skeletal development, we generated a *Ctsk^-/-^/Mmp9*^-/-^ DKO mouse model by crossing *Ctsk*^-/-^ and *Mmp9*^-/-^ mice. The effect of their knockout on protein levels was confirmed with western blotting (Fig. 1a) and genotyping (S. Fig. 1a). We compared long bones from male and female DKO mice to that of WT and the single knockouts (*Ctsk*^-/-^ and *Mmp9*^-/-^) using X-ray analysis (Fig. 1b) which showed that DKO mice continued to display higher femoral density than the WT or *Ctsk^-/-^* mice at 4, 8, and 10 weeks of age (S. Fig. 1b-d), and that the osteopetrosis was progressively exacerbated during postnatal development. DKO mice exhibited severe osteopetrosis, even relative to the osteopetrosis normally observed in *Ctsk*^-/-^ mice (Fig. 1b). Consequently, µCT analysis of distal femurs of 10-week-old mouse showed an increase in bone density in *Ctsk*^-/-^ mice but normal bone density in *Mmp9*^-/-^ mice as compared to WT controls (Fig. 1c and 1d). The femoral sections of WT and *Mmp9*^-/-^ mice at 10 weeks showed similar indices of bone formation, further confirming that *Mmp9* deletion does not affect bone development while *Ctsk^-/-^* mice showed a decrease in trabecular space and structural modal index as compared to WT and *Mmp9^-/-^* mice (Fig. 1d). In addition, the DKO femurs exhibited a 1.5-fold increase in trabecular thickness, 7-fold increase in bone volume/tissue volume, 3-fold decrease in trabecular bone space, 7-fold increase in connectivity density, 2.5-fold increase in trabecular bone number, and a 1.5-fold increase in cortical bone volume/tissue volume compared to WT (Fig. 1d), the serum concentration of carboxy-terminal collagen crosslinks (CTX; bone resorption marker) was not significantly altered in *Mmp9^-/-^* mice, while it decreased by nearly 2-fold and surprisingly by almost 4-fold in 8-wk-old *Ctsk^-/-^ and DKO* mice, respectively (Fig. 1e). We also found total procollagen type 1 N-terminal propeptide (P1NP) level, a bone formation marker, was not altered in *Ctsk^-/-^* or *Mmp9^-/-^*, and only mildly reduced in DKO mice (Fig. 1f), indicating normal bone formation in DKO mice. We performed H&E staining (Fig. 1g) and Von Kossa staining (Fig. 1h) to examine the trabecular bone in the mutant and WT mice at 10 weeks of age. Trabecular bone was comparable between *Mmp9*^-/-^ and WT mice, but markedly increased in *Ctsk*^-/-^. Consistently, DKO mice exhibited the greatest increase in trabecular bone. Collectively, the results indicated that DKO of *Ctsk* and *Mmp9* caused a more severe osteopetrotic phenotype than *Ctsk* deletion, which causes mild osteopetrosis, while *Mmp9* deletion had normal bone density, indicating that Ctsk could compensate for the loss of Mmp9 in *Mmp9*^-/-^ mice.

**Figure 2:**
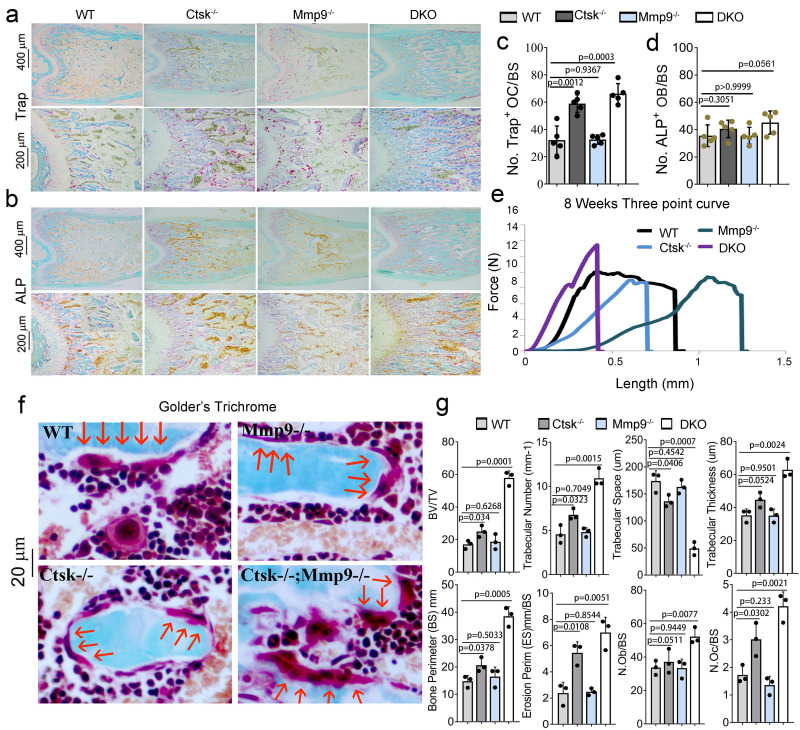
** Osteoclasts from *Ctsk* and *Mmp9* DKO mice showed reduced bone matrix degradation potential and stiff fragile bone. (a)** TRAP staining of WT, *Ctsk^-/-^*, *Mmp9^-/-^*, or *Ctsk^-/-^/ Mmp9^-/-^* mice. **(b)** ALP staining of WT, *Ctsk^-/-^*, *Mmp9^-/-^*, or *Ctsk^-/-^/ Mmp9^-/-^* mice. **(c)** Quantification of TRAP+ cells from **(a)** (n=5 independent views). **(d)** Quantification of ALP+ cells from **(b)** (n=5 independent views). © Flexural test using three point bending of femurs (n=3 animals). **(f)** Goldner's Trichrome analysis of WT or *Ctsk^-/-^/Mmp9^-/-^* femurs at 8 weeks of age. Higher magnification images are shown with OCs indicated by red arrows. **(g)** bone volume/tissue volume, bone mineral density, and other histomorphometric parameters from 8-week-old *Ctsk^-/-^/Mmp9^-/-^* and WT mice femurs (n=4 animals). All the data are presented as mean values ± SD; two-tailed unpaired *t* test; ns. non-significant.

Osteoclasts from *Ctsk*^-/-^/*Mmp9*^-/-^ DKO mice showed reduced bone matrix degradation potential and highly rigid and inflexible bone. To further explore the severe osteopetrosis observed in DKO mice, we examined the number of osteoclasts using TRAP staining (Fig. 2a), and osteoblasts through ALP staining (Fig. 2b) in adult mouse femurs. We observed increases in TRAP+ osteoclasts (Fig. 2a, c) and no significant change in ALP+ osteoblasts in DKO mice (Fig. 2b, d). We used the three-point bend assay to determine the material properties of mouse femurs from WT, *Ctsk^-/-^*, *Mmp9^-/-^*, and DKO (*Ctsk^-/-^*/*Mmp9^-/-^*) mice (Fig. 2e). *Ctsk^-/-^* femurs required substantially less force (comparable to WT) but with similarly narrow deflection prior to breakage. The next to break were WT bones, which had considerably more deflection and much of it at the maximum force required for the bone to break. The *Mmp9^-/-^* bones deflected considerably more than any of the other bones tested but also required a good deal of force to break, like the WT bones. Surprisingly, we found that the DKO femurs broke with the least amount of deflection, yet required the most force to increase deflection, indicative of highly rigid but inflexible bone. Ultimately, these combine to indicate that while *Ctsk^-/-^* and *Mmp9^-/-^* have separate and distinct effects on bone's mechanical characteristics, when combined they result in highly rigid bone that breaks at significantly higher force than single mutants alone, making the bone more rigid than either of the mutations alone. We confirmed changes in the numbers of large multinucleated osteoclasts using Goldner's Trichrome staining (Fig. 2f) followed by histomorphometry analysis (Fig. 2g). Quantification of the bone characteristics showed a 3.5-fold increase in BV/TV, 2.5-fold increase in trabecular number, 4-fold decrease in trabecular space, 1.5-fold increase in trabecular thickness, 2.5-fold increase in bone perimeter, 2.5-fold increase in erosion perimeter, 1.3-fold increase in osteoblasts/bone perimeter, and a 3-fold increase in osteoclasts/bone perimeter in DKO compared to WT mice (Fig. 2g).

**Figure 3:**
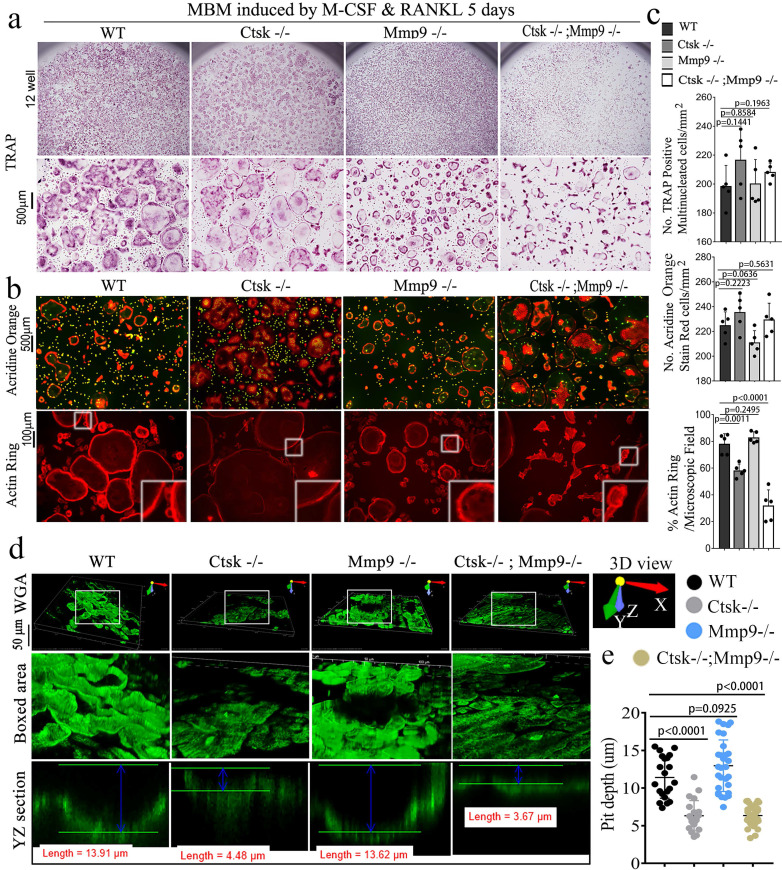
** The double deletion of *Ctsk* and *Mmp9* impairs osteoclast function *in vitro*.** (**a**) TRAP staining shows decreased OC formation in MBM cells isolated from *Mmp9^-/-^,Ctsk^-/-^ and Ctsk^-/-^/ Mmp9^-/-^* cells that were stimulated by M-CSF and RANKL for 5 days. Higher magnification images are shown in the lower panels. **(b)** MBM cells isolated from WT, *Ctsk^-/-^*, *Mmp9^-/-^*, or *Ctsk^-/-^/ Mmp9^-/-^* mice and then treated with M-CSF and RANKL for 5 days to promote OC differentiation.** (b, Top panel)** Acridine orange staining revealed that extracellular acidity (red-orange) was significantly reduced in DKO cells. **(b, Bottom panel)** F-actin ring formation assay for cell cultures shows disrupted or absent ringed structures of F-actin dots (actin rings) in *Ctsk^-/-^/Mmp9^-/-^*and *Ctsk*^-/-^ mice. Inset is the magnified image of the boxed areas. **(c)** Quantification for panels A and B (n=5 independent experiments). **(d)** Pre-OC seeded on bone slices were cultured with M-CSF and RANKL for 6 days before being submitted to bone resorption analysis. WGA-FITC staining revealed a drastic decreased in bone resorption in *Ctsk^-/-^* OCs and a near absence of bone resorption in *Ctsk^-/-^ Mmp9^-/-^* OCs, but *Mmp9^-/-^* and WT cells showed similar bone resorption abilitie©(**e**) Quantification for D is shown (n>20, Pit depth). All the data are presented as mean values ± SD; two-tailed unpaired *t* test; ns. non-significant.

**The double deletion of *Ctsk* and *Mmp9* significantly impairs osteoclast function *in vitro*.** We next investigated the effect of *Ctsk/Mmp9* deletion on OC formation and activity *in vitro*. Mouse bone marrow (MBM) cells isolated from WT, *Ctsk*^-/-^, *Mmp9*^-/-^, and DKO mice were stimulated with M-CSF and RANKL for 5 days to promote OC formation (Fig. 3). *Ctsk* deletion showed no overt effect on OC formation and OC size (Fig. 3a, c) as compared to WT controls. Further, *Mmp9* deletion did not affect OC number, but OCs appeared to be smaller than those of WT and *Ctsk^-/-^* (Fig. 3a, c). *Ctsk^-/-^/Mmp9^-/-^* OCs appeared to be larger than *Mmp9^-/-^* OCs, suggesting than Ctsk could compensate for the lack of *Mmp9* in *Mmp9^-/-^* mice by increasing OC size. We next examined the effects of *Ctsk* and *Mmp9* deficiencies on extracellular acidification (Fig. 3b, top panel), a process that is critical for actin ring formation (Fig. 3b, bottom panel). To test the effects of knockouts on osteoclast function, cultures were made. The culture was submitted to acridine orange staining for analysis of extracellular cellular acidification (Fig. 3b, top panel) or rhodamine phalloidin staining for analysis of actin ring formation (Fig. 3b, bottom panel). MBM cells lacking *Ctsk* or *Mmp9* exhibited normal acidification, but DKO drastically abrogated acidification by mature OCs (Fig. 3b, c). Consistently, *Ctsk^-/-^* and *Ctsk^-/-^/Mmp9^-/-^* OCs were significantly smaller than WT and *Mmp9^-/-^* OCs, indicating that lack of *Ctsk* and *Mmp9* together altered terminal OC differentiation. Moreover, actin ring formation was severely defective in DKO OCs (Fig. 3b, c). Consistent with our *in vivo* results, our bone resorption analysis showed that *Mmp9* deficiency showed no overt effect on bone resorption (Fig. 3d, e) whereas *Ctsk* deletion showed a 5-fold reduction in bone resorption, and DKO caused a 7-fold reduction in bone resorption (Fig. 3d, e). Collectively, the data indicated that DKO of Ctsk and Mmp9 had a significant impact on OC maturity and activity compared to the single deletion. Furthermore, the data supported our hypothesis that Ctsk compensates for loss of Mmp9 in alleviating the effects of the gene deletion on OC maturity and activity.

**Figure 4:**
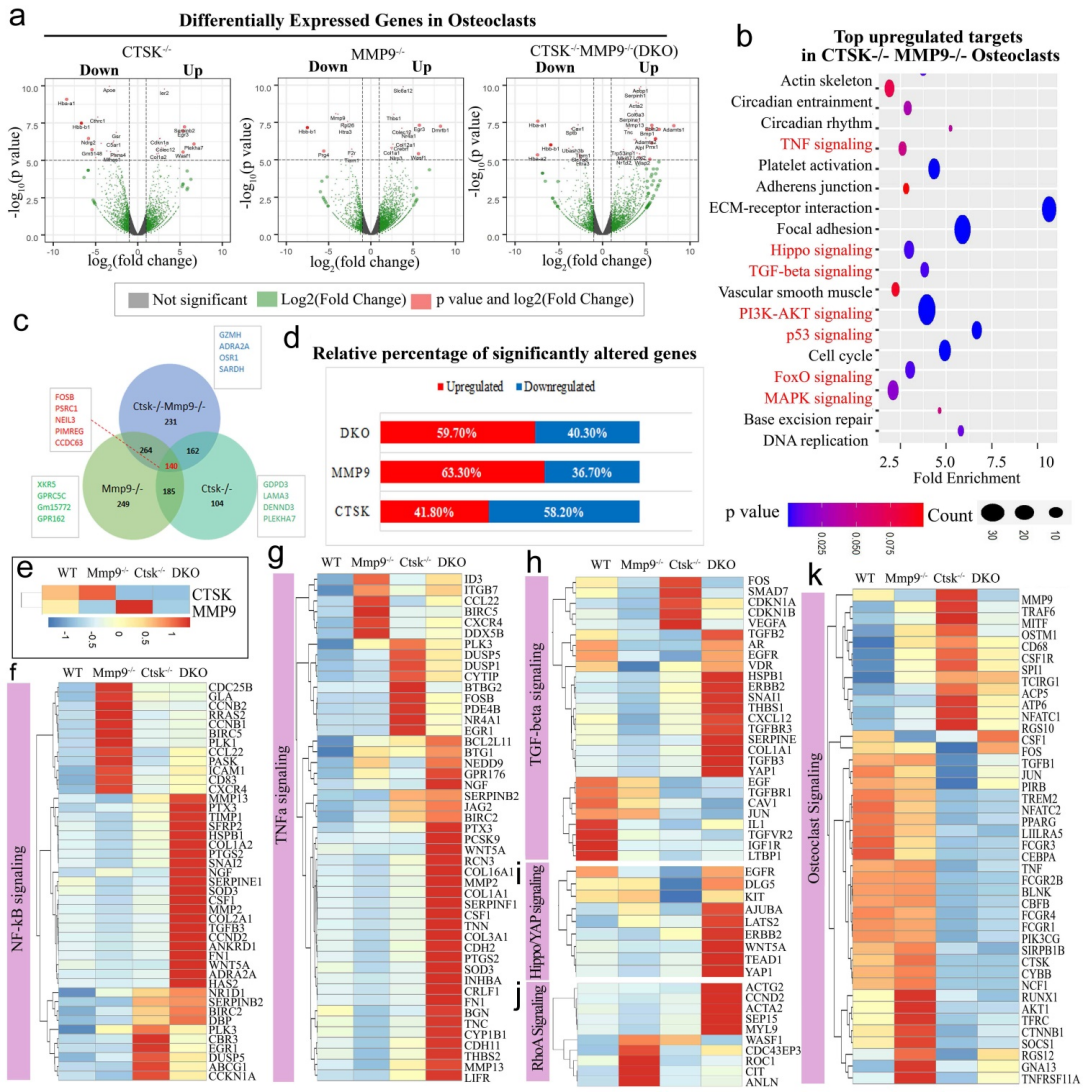
** RNA-seq analysis of *Ctsk^-/-^*,* Mmp9^-/-^, and Ctsk^-/-^/Mmp9^-/-^* osteoclasts showed their distinct functions in regulating genes' expression and signaling pathway activation. (a)** A volcano plot illustrating differentially regulated gene expression from RNA-seq analysis between the control *Ctsk^-/-^, Mmp9^-/-^,* and *Ctsk^-/-^/Mmp9^-/-^* osteoclasts. Values are presented as the log2 of tag counts. **(b)** Top upregulated signaling pathways in DKO osteoclasts analyzed by KEGG database. **(c)** Venn diagram showing the total number of differentially expressed genes (P < 0.05) between *Ctsk^-/-^/Mmp9^-/-^*, *Ctsk^-/-^, and Mmp9^-/-^* mice. **(d)** RNA-seq comparison revealed the percentage of upregulated versus downregulated genes a total of 22669 genes expressed in respective mouse g©p. **(e)** Heatmap to confirm successful knockout of Ctsk and Mmp9. **(f-k)** Heatmaps of representative signaling pathways NF-κB, TNFα, TGF-beta, Hippo/YAP, RhoA, and osteoclast signaling in *Ctsk^-/-^/Mmp9^-/-^*, *Ctsk^-/-^, and/or Mmp9^-/-^ mice* osteoclasts.

RNA-seq analysis of *Ctsk^-/-^*,* Mmp9^-/-^ and Ctsk^-/-^/Mmp9^-/-^* osteoclasts showed their distinct functions in regulating genes' expression and signaling pathway activation. Using unbiased transcriptome-wide RNA-seq data from *Ctsk^-/-^, Mmp9^-/-^,* and* Ctsk^-/-^/Mmp9^-/-^* osteoclasts, we then examined *Ctsk^-/-^* and *Mmp9^-/-^* mediated transcriptional targets that could account for osteoclast function defects (Fig. 4a). Quantification data (S. Fig. 2) from volcano plots in Fig. 4a show differentially regulated gene expression from RNA-seq analysis between the controls, single KO, and DKO mice osteoclasts. Notable genes upregulated in the DKO osteoclasts include Wisp2, Aebp1, and Mmp13. Wisp2 encodes a member of the Wnt1 inducible signalling pathway (WISP) protein subfamily, and this gene may be downstream in the Wnt1 signalling pathway 47. Mmp13 is a major enzyme that targets cartilage for degradation 48. Interestingly, both *Ctsk^-/-^* and *Mmp9^-/-^* osteoclasts have increased expression of Egr3, Wasf1, Colec12, and Col1a2, indicating overlapping function of Mmp9 and Ctsk in osteoclasts. However, DKO osteoclasts showed unique patterns of gene expression that could not be explained by overlapping functions of Ctsk and Mmp9, which caused us to speculate that some gene expression changes could be due to changes at the epigenetic level. Notably, Nr1d2 (a transcriptional repressor), Loxl2 (mediates the post-translational oxidative deamination), and Prrx1 (transcription co-activator) were all upregulated in DKO osteoclasts but not Ctsk KO or Mmp9 KO osteoclasts. Thus, combined deletion of Ctsk and Mmp9 could lead to epigenetic changes that are responsible for the unique phenotype of DKO mice. KEGG enrichment analysis was used to determine the most significantly affected categories of genes that were upregulated with double* Ctsk* and* Mmp9* knockout, as well as* Ctsk* KO, and *Mmp9* KO, relative to WT (Fig. 4b, S. Fig. 3). The top GO upregulated categories were selected according to the P-values and enrichment score and illustrated as number of genes upregulated in respective category. Consistent with our previous results, among the top upregulated gene clusters were associated with Tnf, Hippo, Tgf-beta, PI3K-Akt, p53 signalling pathways (Fig. 4b).

We then examined the total number of differentially expressed genes (p < 0.05) and overlaps between *Ctsk^-/-^/Mmp9^-/-^*, *Ctsk^-/-^, and Mmp9^-/-^* mice and found that 140 differentially expressed genes overlapped between the three groups, including Fosb, Psrc1, Neil3, Pimreg, and Ccdc63 (Fig. 4c). Among these significantly differentially expressed genes, transcripts of 58.2% genes were downregulated in *Ctsk^-/-^* osteoclasts, 36.7% were downregulated in *Mmp9^-/-^* osteoclasts, and 40.30% were downregulated in *Ctsk^-/-^/Mmp9^-/-^* osteoclasts compared to control (Fig. 4d). In gene expression analysis, efficient knockout of Ctsk and/or Mmp9 was confirmed (Fig. 4e). Heatmaps of representative genes demonstrated that the many genes in the Nf-κB, Tnf-α, Tgf-beta, Hippo/Yap, and RhoA, signalling pathways were significantly upregulated in *Ctsk^-/-^/Mmp9^-/-^* mice osteoclasts compared to the control osteoclasts (Fig. 4e-j). Interestingly, we found variable differential expressions of genes important to osteoclast differentiation, activation, and function (Fig. 4k). *Ctsk^-/-^* osteoclasts have significantly increased expression of Traf6, Mitf, Ostm1, CD68, Csf1r, Spi1 (Pu.1), Tcirg1, Acp5, Nfatc1, and Rgs10, while expression of Cebpa, Runx1, Akt1 were markedly downregulated in both *Ctsk^-/-^* and DKO osteoclasts (Fig. 4k). These results showed that inflammation-related signalling pathway gene expression were significantly upregulated by Ctsk and Mmp9 deficiency and Ctsk and Mmp9 may act as crucial regulators in osteoclasts homeostasis.

**Figure 5:**
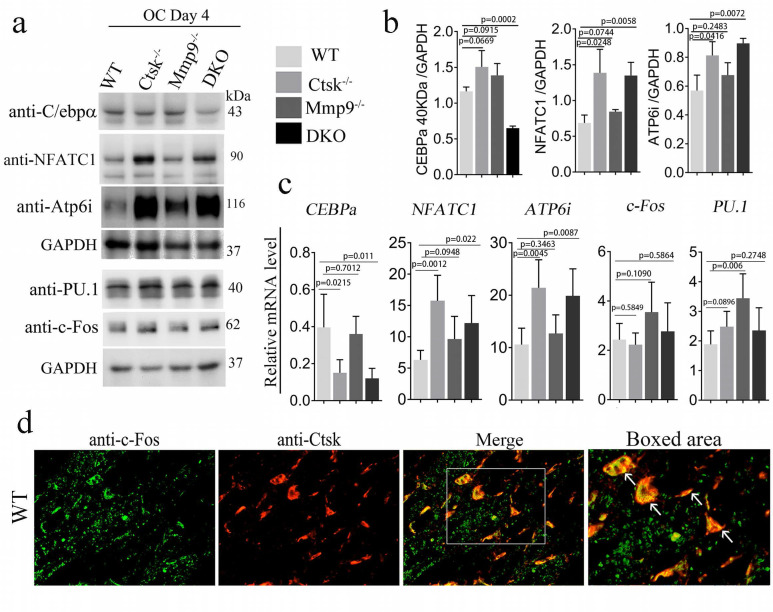
***Ctsk^-/-^/Mmp9^-/-^* osteoclasts exhibited low Cebpa expression and high Nfatc1 expression, and Ctsk is co-localized to the cell nucleus along with c-Fos. (a)** Western blots for important transcriptional factors in osteoclast at the end of 4-day osteoclastogenesis induction, normalized to GAPDH. **(b)** Quantification of A (n=3 independent experiments). **(c)** qPCR to assess mRNA expression of important transcriptional factors in osteoclast formation and function at the end of 4-day osteoclastogenesis induction, normalized to Hprt (n=3 independent experiments). **(d)** Immunofluorescent staining (IF) of c-Fos (green) in addition to Ctsk (red) in mouse femur. Merged image (yellow) indicated co-localization of the proteins (n=3 independent experiments). All the data are presented as mean values ± SD; two-tailed unpaired *t* test; ns. non-significant.

*Ctsk^-/-^/Mmp9^-/-^* osteoclasts exhibited low C/EBPα expression and high Nfatc1 expression, and Ctsk is co-localized to the cell nucleus along with c-Fos. We sought to further examine the molecular basis underlying the dramatic changes in DKO osteoclast function. As we further examined the expression levels of the transcription factors that are important to osteoclast differentiation and function, we found lower protein levels of Cebpα, a regulator of osteoclast differentiation in DKO mice (Fig. 5a, b). Interestingly, Pu.1 and Atp6i expression increased in all 3 mutant groups, c-fos increased in *Mmp9^-/-^* and DKO while Nfatc1 protein was significantly increased in *Ctsk^-/-^* and DKO but was normal in *Mmp9^-/-^* osteoclasts (Fig. 5b, c). To determine whether Ctsk could translocate to the cell nucleus, we performed immunofluorescent staining to detect co-localization of Ctsk and c-Fos, which is an important osteoclast transcription factor located in the nucleus 49. Immunofluorescent staining revealed that Ctsk may co-localize to the cell nucleus along with important osteoclast transcription factor c-Fos, as denoted by the yellow areas in merged images (Fig. 5d). These findings suggest that Ctsk and Mmp9 act as important osteoclast gene expression regulators.

**Figure 6:**
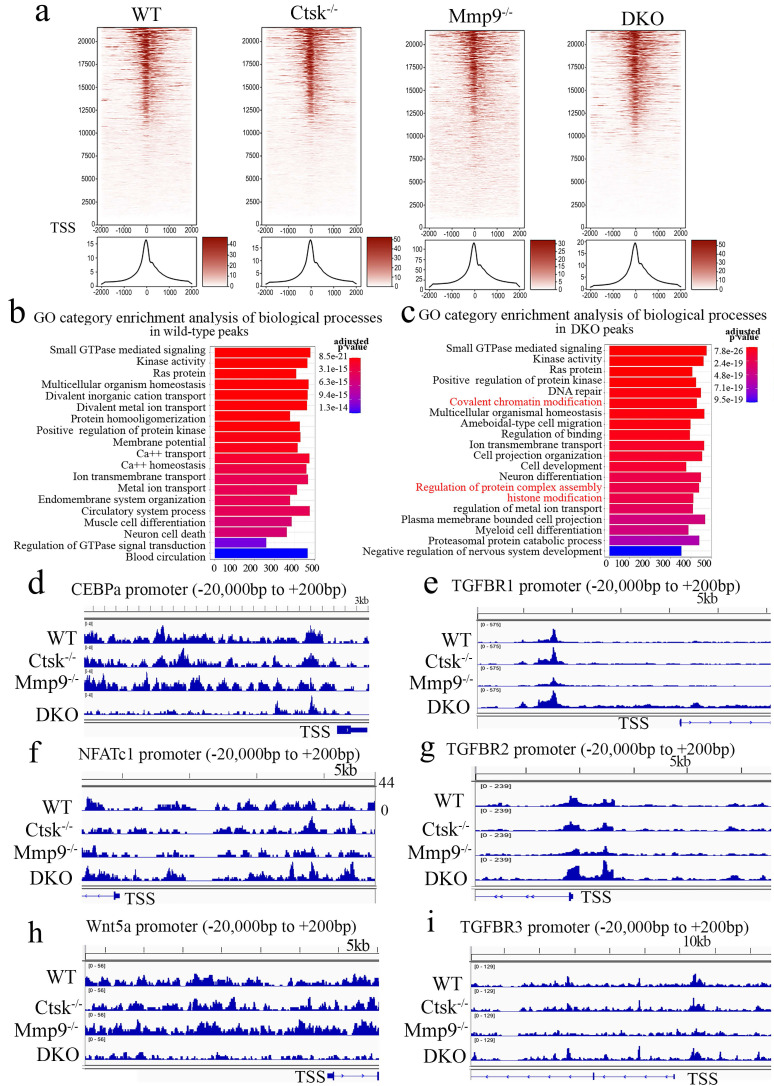
** ATAC-Seq Profiling of WT, *Ctsk^-/-^*, Mmp9^-/-^ and DKO osteoclasts. (a)** Heat maps (top) and average plots (bottom) of ATAC-seq signals indicating transposase hypersensitive sites (THS) in WT, *Ctsk^-/-^*, *Mmp9^-/-^* and DKO osteoclasts with each genotype having its own panels for comparison. The genes in the heat maps (top panel) are displayed in rows ranked from highest signal/most openness (top) to least (bottom), with “heat” indicating open regions (as determined by ATAC-seq reads) around the TSS (-2000bp on the left to +2000bp on the right). The average plot (bottom panels) displays average openness at sites around the TSS as a histogram of openness around the TSS (-2000bp on the left to +2000bp on the right). **(b, c)** GO category enrichment analysis of biological process in wild-type (**b**) and *Ctsk^-/-^/Mmp9^-/-^* DKO osteoclasts (**c**). Each bar in the chart corresponds to a significantly enriched GO biological process gene group, relative to an unbiased/random selection of genes. Colors of the bars indicate the p-value of the enrichment. The length of the bars indicated the number of genes included in the ATAC-seq groups. In (**c**) clusters of interest are highlighted in red. **(d-i)** Peak chart comparison of ATAC-seq data along promoter regions of important genes of interests in WT, *Ctsk^-/-^*, *Mmp9^-/-^* and DKO. X-axis locations correspond to locations along the promoter region of the marked genes, and Y-axis bar height indicated the intensity of signal at the locations on the genome.

**ATAC-Seq Profiling of WT, *Cts****k^-/-^***, *Mmp9^-/-^* and DKO osteoclasts.** To further examine and validate the mechanisms underlying observed changes in gene expression using an unbiased genome-wide approach, ATAC-Seq profiling was performed using WT, *Ctsk^-/-^*, *Mmp9^-/-^,* and DKO osteoclasts. Average plots and heat maps were used to show transposase hypersensitive sites (THS) around known transcription start sites (TSS) in WT, *Ctsk^-/-^*, *Mmp9^-/-^* and DKO osteoclasts, with evidence indicating that open chromatin is enriched close to TSS which makes the results in line with other functional ATAC-seq results (Fig. 6a). There appears to be a slight increase in THSs in *Mmp9^-/-^* osteoclasts, but generally the distinctions between open chromatin regions are small (Fig. 6a). Distribution of ATAC-seq, relative to genomic features, shows that our ATAC-seq found considerable open chromatin in many distinct regions of the genome, especially introns, distal intergenic regions, and proximal promoters (<1kb from the TSS) (S. Fig. 4). Open chromatin is particularly enriched in the promoter regions as well as introns, indicating that the results are legitimate in DKO samples (S. Fig. 4), with similar results in WT and single knockouts (S. Fig. 4). There is an increase in THS regions in the distil intergenic spaces in Mmp9^-/-^ osteoclasts perhaps indicating increased background, especially in combination with higher signal in Fig. 6a. Further, GO category enrichment analysis of biological process revealed several changes in chromatin openness in categories of genes of interest in WT (Fig. 6b) and DKO (Fig. 6c) osteoclasts, with changes observed between them. For instance, there was an enrichment of chromatin openness near genes involved in covalent chromatin modifications as well as histone modification in DKO osteoclasts (Fig. 6c). This indicated that these important processes in epigenetic regulation are changed in DKO osteoclasts where these enrichments were not observed in WT osteoclasts, although it does not indicate whether these regions are being targeted by inhibitors or activators (Fig. 6b). Together this indicated the potential for secondary effects of DKO on epigenetic regulation. Finally, we sought to examine chromatin openness in the promoters of several genes implicated in osteoclast formation and function as well as those involved in bone homeostasis (Fig. 6d-i). Comparison of ATAC-seq data along promoter regions of important genes of interests revealed deficiency of Ctsk and DKO led to reduced chromatin accessibility in the promoter regions of Cebpa (Fig. 6d) and Wnt5a (Fig. 6h), while increased in Nfatc1 (Fig. 6f) and Tgfbr genes (Fig. 6e, g, i). Interestingly, the transposase accessibility along the promoter region of Cebpa was not changed in single knock-out *Ctsk^-/-^* and *Mmp9^-/-^* osteoclasts compared with WT OCs, but the gene accessibility along the promoter region of Cebpa in the DKO OCs was significantly reduced (Fig. 6d), suggesting a potential role of both Ctsk and Mmp9 as epigenetic regulators of Cebpa promoter activity, and indicating that they may have redundant roles in regulating chromatin accessibility, as they have comparable protease activities. In summary, ATAC-Seq profiling indicated changes in chromatin openness in DKO osteoclasts compared to WT, *Ctsk^-/-^*and *Mmp9^-/-^* osteoclasts, which led to reduced access to the promoter region of the *Cebpa* gene and increased access to the promoter regions of Nfatc1 and Tgfbr genes.

**Figure 7:**
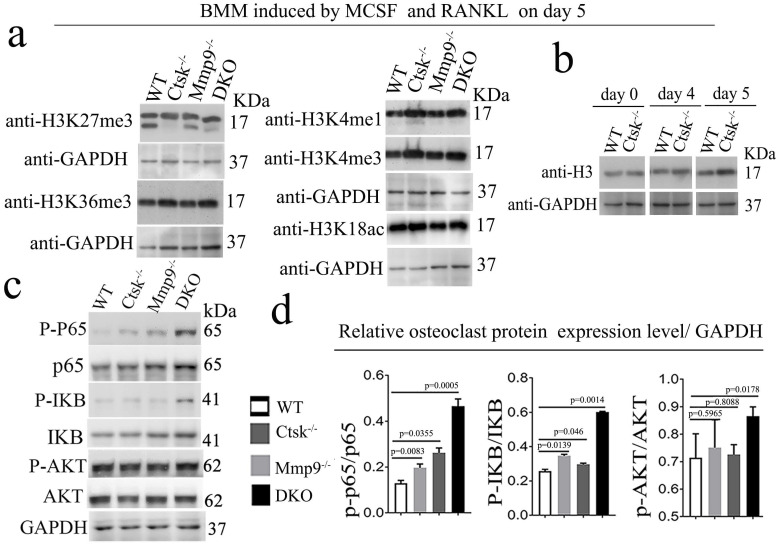
**Ctsk and Mmp9 may regulate gene expression through targeted proteolysis of H3K27me3. (a)** Western blots to examine the expression level of histone H3 epigenetic modifications (H3K27me3, H3K36me3, H3K4me1, H3K4me3, H3K18ac) at day 5 of osteoclastogenesis induction, normalized to GAPDH (n=3 independent experiments). **(b)** Western blots to examine the expression level of histone H3 (n=3 independent experiments). **(c)** Western blots for important transcriptional factors in osteoclast at the end of 4-day osteoclastogenesis induction, normalized to GAPDH. **(d)** Quantification of C (n=5 independent experiments). All the data are presented as mean values ± SD; two-tailed unpaired *t* test; ns. non-significant.

**Ctsk and Mmp9 may regulate gene expression through targeted proteolysis of H3K27me3.** Kim et al. have reported Mmp9 facilitates selective proteolysis of the histone H3 tail at genes necessary for proficient osteoclastogenesis, suggesting potential role of epigenetic regulation, however the pathomechansim has not yet been explored 14. We examined methylation levels of H3K27 (Fig. 7a, b), and to this end, we found dynamic cleavage of H3K27me3 at various duration of osteoclast differentiation induction (Fig. 7a, b). Compared to the cleaved H3K27me3 in WT and Mmp9 KO, the absence of H3K27me3 cleavage in *Ctsk^-/-^* osteoclast suggests that Ctsk may be responsible for H3K27me3 cleavage. Conversely, *Mmp9^-/-^* osteoclasts still had H3K27me3, therefore, Mmp9 might not be involved in cleavage of this protein. H3K36me3 and H3K4me1/3 expression levels are unchanged (Fig. 7a, b). The epigenetic changes might affect important osteoclast regulators such as Nfatc1. Interestingly, we found that upon RANKL stimulation, there was increased activation of NF-kB p65 and IKB in DKO osteoclasts, while Akt was only mildly elevated in DKO osteoclasts (Fig. 7c, d). Taken together, our data indicated that Ctsk and Mmp9 can translocate to the osteoclast nucleus and may play a role in regulating epigenetic modifications thereby affecting osteoclast activation and osteoclast function.

**Figure 8:**
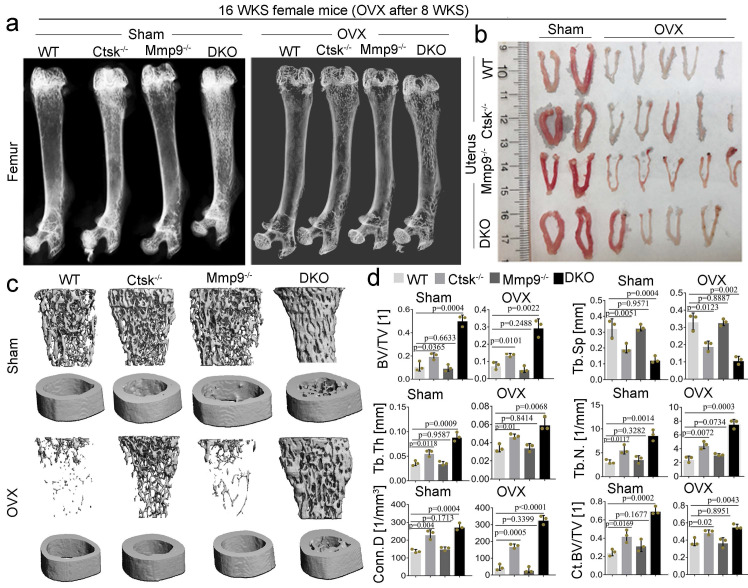
***Ctsk* and *Mmp9* double knockout protects against ovariectomy induced bone loss. (a)** Representative images of radiographic analysis of mouse femurs (n=7 animals). **(b)** Assessment of uterine size to confirm the effects of OVX (n=7 animals). **(c)** uCT analysis of WT, *Ctsk^-/-^*, *Mmp9^-/-^* and DKO femurs with or without OVX procedure (n=3 animals). **(d)** Quantification of bone morphometry from c. All the data are presented as mean values ± SD; two-tailed unpaired *t* test; ns. non-significant.

***Ctsk* and *Mmp9* double knockout protects against ovariectomy induced bone loss.** To determine whether dual targeting of Mmp9 and Ctsk that could be leveraged to block known inflammation-related bone loss, 2-month-old female mice was used in the ovariectomy experiments. we assessed the effects of ovariectomy (OVX) on bone in our DKO, and single knock out, 4-month-old mouse models (Fig. 8; OVX validated in Fig. 8b). We found that while *Ctsk^-/-^* protected against OVX mediated osteoporosis, the DKO's effect on blocking osteoporosis was significantly stronger (Fig. 8a and c), with a striking increase in bone volume relative to tissue volume, decreases in trabecular bone space, and increases in connectivity density, in line with previous observations of the osteopetrosis in DKO mice (Fig. 8d). Together, these findings indicate that dual targeting of Mmp9 and Ctsk may be useful for blocking osteoporosis induced bone loss.

## Discussion

**We found that Ctsk may play a novel role as an epigenetic regulator of osteoclast gene expression, activation, and function.** Our work showed that *Ctsk^-/-^/Mmp9^-/-^* (Double-knockout or DKO) mice have a striking phenotype with markedly increased bone density and rigidity, suggesting that Ctsk and Mmp9 play an important role in osteoclasts. Transcriptome analysis using RNA-sequencing of *Ctsk^-/-^ /Mmp9^-/-^* osteoclasts showed that DKO promotes Nf-κB and Tnf-α signaling pathway activation. Importantly, we revealed Ctsk co-localized to the cell nucleus along with important osteoclast transcription factor c-Fos. While ATAC-seq data revealed *Ctsk^-/-^/Mmp9^-/-^* DKO led to dramatically reduced transcriptional activity in the promoter region of Cebpa, indicating that the reduced OC gene expression in DKO OCs could be mediated through epigenetic regulation of the Cebpa promoter. These results indicate that Ctsk may play novel functions as epigenetic regulators in osteoclast gene expression, activation, and bone resorption. Given complex control of osteoclast function, not only does Ctsk function as the effector enzyme, but may play critical roles in the regulatory network by cleaving key nuclear proteins, which could alter the expression of osteoclast differentiation and functional genes.

**Our data demonstrated a potential role of Ctsk in regulating the stability of H3K27me3.** Gene expression changes in* Ctsk^-/-^, Mmp9^-/-^,* and *Ctsk^-/-^/Mmp9^-/-^* osteoclasts cannot be explained by the deficiency of Ctsk and Mmp9 in terms of the traditionally thought of roles of these genes in osteoclasts, because they are not known for gene expression regulation, and especially in vitro where their function breaking-down extracellular bone matrix might result in the release of cell signaling factors. Our data indicated that Ctsk can translocate to the osteoclast nucleus, indicating its potential role in regulating epigenetic modifications thereby affecting osteoclast gene expression. Surprisingly, we revealed that Ctsk may regulate the stability of H3K27me3. Previous studies have revealed that Mmp9 facilitates selective proteolysis of the histone H3 tail at genes necessary for proficient osteoclastogenesis 14. Our study is the first to demonstrate to demonstrate a potential role of Ctsk to regulate the stability of H3K27me3 and underscores a potential role of epigenetic regulation in mediating osteoclastogenesis and osteoclast function as evident by the gene expression profiles in the Ctsk and Mmp9 DKO mouse model. Although the potential mechanism underlying how Ctsk cleaves H3K27me3 remains to be explored.

**The complementary function of Ctsk and Mmp9 in epigenetic gene expression regulation.** Notably, in genome-wide RNA-seq studies, many genes in the Nf-κB, Tnf-α, Tgf-beta, Hippo/Yap, and RhoA, signalling pathways were significantly upregulated in *DKO* osteoclasts, with differential expressions of genes important to osteoclast differentiation, activation, and function (Fig. 4). Specifically, the expression of Cebpa, Runx1, and Akt1 were markedly downregulated and Nfatc1 was upregulated in DKO osteoclasts. Cebpa is a known regulator of osteoclast function 18, Runx1 is a central regulator of osteogenesis for bone homeostasis 37, while Akt1 plays a critical role in osteoclast differentiation 50. In addition, analysis of upregulated and downregulated genes expression in *Mmp9^-/-^*,* Ctsk^-/-^*, and DKO osteoclasts suggested a complementary function of Ctsk and Mmp9, as shown by compensatory genes expression in the single mutants. For instance, both *Mmp9^-/-^* and *Ctsk^-/-^* osteoclasts have increased expression of Egr3, Wasf1, Colec12, and Col1a2 (Fig. 4), indicating overlapping function of Mmp9 and Ctsk in osteoclasts. The changes in accessibility of promoters for Cebpa , Tgfbr1, Nfatc1, and Tgfbr2 indicate an increase in access to the promoters of the Nfatc1 and Tgfbr genes and reduced access to the promoters of Cebpa, transcription factors involved in osteoclast differentiation (Fig. 6), 39, and Wnt5a, its pathway is crucial for osteoclastogenesis in physiological and pathological environments 51. This could be indicative of a change in epigenetics mediated by the proteases Ctsk and Mmp9, which could be related to the cleavage of histones to remove epigenetic marks 14, 52, 53. Interestingly, a recent study revealed that gene expression is correlated with H3K27me3 levels during mouse embryonic stem cell division induced by Wnt3a 22. While Zhu et al. recently showed that histone modification of H2AK119ub1 and H3K27me3 show a unique genome-wide uncoupling phenomenon during early mouse embryonic development 23. Our study has demonstrated that DKO osteoclasts showed unique patterns of gene expression that further confirmed a complementary function of Ctsk and Mmp9 at the level of epigenetic gene expression regulation. While Nfatc1 is required for osteoclast differentiation, Cebpa is the regulator of osteoclast function, which explains why DKO resulted in defective osteoclast activation.

**The functions of Ctsk as an epigenetic regulators maintains bone homeostasis by orchestrating multiple signaling pathways**. Cathepsins from lysosomal leakage that lack secretory signals have also been shown to translocate to the cell nucleus and mediate proteolytic activation of transcription factors, which play a regulatory role in cell differentiation and proliferation 12, 13, 15. Histone modifications and the associated proteases can be altered dynamically throughout the cell cycle. Compared to the cleaved H3K27me3 in WT, the absence of H3K27me3 cleavage in *Ctsk^-/-^* osteoclast suggests that Ctsk may be responsible, either directly or indirectly, for H3K27me3 cleavage. Conversely, *Mmp9^-/-^* osteoclasts still had uncleaved H3K27me3. The lack of H3K27me3 cleavage in DKO osteoclasts could be because of the changes of OC gene expression in DKO OCs. H3K27me3 is associated with the downregulation of nearby genes via the formation of heterochromatic regions 54. The dramatic changes of OC gene expression and increased bone density in DKO OCs indicated that there may be complementary and unique functions of Mmp9 and Ctsk as epigenetic regulators for maintaining bone homeostasis by orchestrating multiple signaling pathways as shown in Figure 4 and 6. The mechanism underlying the complementary functions remain to be explored. Interestingly, we found that there were no changes in H3K4me3, H3K36me3, and H3K4me1 protein levels in DKO OCs, indicating that Ctsk may specifically regulate OC gene expression through H3K27me3.

Loss of bone resorption function and impaired activation in DKO osteoclasts may result from reduced Cebpa expression and increased Nfatc1 expression. Our previous works showed that Cebpa plays critical role osteoclast lineage commitment 16, 21, and osteoclast terminal differentiation and activation 17, 18, 21. Our present study demonstrated Ctsk can translocate to the osteoclast nucleus, with significantly altered protein levels of several osteoclast transcription factors such as Cebpa and Nfatc1. Notably, ATAC-seq data revealed *Ctsk^-/-^/Mmp9^-/-^* DKO led to dramatically reduced transcriptional activity in the promoter region of Cebpa, indicating that the reduced OC gene expression in DKO OCs could be mediated through epigenetic regulation of the Cebpa promoter. Cebpa is critical for osteoclast differentiation by regulating OC lineage commitment and is also important for OC differentiation and function, and importantly we have recently revealed that Cebpa controls osteoclast terminal differentiation, activation, function, and postnatal bone homeostasis through direct regulation of Nfatc1 17, 18. While Nfatc1 is required for osteoclast differentiation, Cebpa is the regulator of osteoclast function, which explains why DKO impaired osteoclast function. In addition, we found that upon RANKL stimulation, there was increased activation of NF-kB p65 and IKB in DKO osteoclasts, while Akt was only mildly elevated in DKO osteoclasts. NF-kB functions primarily to promote osteoclastogenesis, yet our phenotypic analysis shows enhanced bone formation in DKO mice, which could suggest that there is a negative feedback loop.

In conclusion, we demonstrated that deletion of *Ctsk* and *Mmp9* results in a severe osteopetrosis from impaired OC activation and function due to the loss of the compensatory functions of Ctsk and Mmp9 in osteoclasts. Importantly, we demonstrated that Ctsk is important in osteoclast activation through its roles as potential cleavage enzymes in the cell nucleus and subsequent regulation of gene expression potentially mediated by epigenetic regulation of the osteoclast gene expression. Our study indicated that the enzymes Ctsk and Mmp9 might be instrumental in epigenetic alterations in osteoclast activation and function, and that targeting Ctsk and Mmp9 simultaneously could be an effective therapeutic approach for osteoclastic bone diseases.

## Supplementary Material

Supplementary figures and table.

## References

[1] Kurotaki D, Yoshida H, Tamura T (2020). Epigenetic and transcriptional regulation of osteoclast differentiation. Bone.

[2] Leboime A, Confavreux CB, Mehsen N, Paccou J, David C, Roux C (2010). Osteoporosis and mortality. Joint Bone Spine.

[3] Li YP, Alexander M, Wucherpfennig AL, Yelick P, Chen W, Stashenko P (1995). Cloning and complete coding sequence of a novel human cathepsin expressed in giant cells of osteoclastomas. Journal of bone and mineral research: the official journal of the American Society for Bone and Mineral Research.

[4] Chen W, Yang S, Abe Y, Li M, Wang Y, Shao J (2007). Novel pycnodysostosis mouse model uncovers cathepsin K function as a potential regulator of osteoclast apoptosis and senescence. Human molecular genetics.

[5] Wucherpfennig AL, Li YP, Stetler-Stevenson WG, Rosenberg AE, Stashenko P (1994). Expression of 92 kD type IV collagenase/gelatinase B in human osteoclasts. Journal of bone and mineral research: the official journal of the American Society for Bone and Mineral Research.

[6] Colnot C, Thompson Z, Miclau T, Werb Z, Helms JA (2003). Altered fracture repair in the absence of MMP9. Development (Cambridge, England).

[7] Garnero P, Borel O, Byrjalsen I, Ferreras M, Drake FH, McQueney MS (1998). The collagenolytic activity of cathepsin K is unique among mammalian proteinases. J Biol Chem.

[8] Vu TH, Shipley JM, Bergers G, Berger JE, Helms JA, Hanahan D (1998). MMP-9/gelatinase B is a key regulator of growth plate angiogenesis and apoptosis of hypertrophic chondrocytes. Cell.

[9] Chan CK, Seo EY, Chen JY, Lo D, McArdle A, Sinha R (2015). Identification and specification of the mouse skeletal stem cell. Cell.

[10] Romeo SG, Alawi KM, Rodrigues J, Singh A, Kusumbe AP, Ramasamy SK (2019). Endothelial proteolytic activity and interaction with non-resorbing osteoclasts mediate bone elongation. Nat Cell Biol.

[11] Cackowski FC, Anderson JL, Patrene KD, Choksi RJ, Shapiro SD, Windle JJ (2010). Osteoclasts are important for bone angiogenesis. Blood.

[12] Chapman HA (2004). Cathepsins as transcriptional activators?. Developmental cell.

[13] Goulet B, Baruch A, Moon NS, Poirier M, Sansregret LL, Erickson A (2004). A cathepsin L isoform that is devoid of a signal peptide localizes to the nucleus in S phase and processes the CDP/Cux transcription factor. Molecular cell.

[14] Kim K, Punj V, Kim JM, Lee S, Ulmer TS, Lu W (2016). MMP-9 facilitates selective proteolysis of the histone H3 tail at genes necessary for proficient osteoclastogenesis. Genes & development.

[15] Soond SM, Kozhevnikova MV, Frolova AS, Savvateeva LV, Plotnikov EY, Townsend PA (2019). Lost or Forgotten: The nuclear cathepsin protein isoforms in cancer. Cancer letters.

[16] Chen W, Zhu G, Hao L, Wu M, Ci H, Li YP (2013). C/EBPα regulates osteoclast lineage commitment. Proc Natl Acad Sci U S A.

[17] Chen W, Zhu G, Jules J, Nguyen D, Li YP (2018). Monocyte-Specific Knockout of C/ebpα Results in Osteopetrosis Phenotype, Blocks Bone Loss in Ovariectomized Mice, and Reveals an Important Function of C/ebpα in Osteoclast Differentiation and Function. Journal of bone and mineral research: the official journal of the American Society for Bone and Mineral Research.

[18] Chen W, Zhu G, Tang J, Zhou HD, Li YP (2018). C/ebpα controls osteoclast terminal differentiation, activation, function, and postnatal bone homeostasis through direct regulation of Nfatc1. J Pathol.

[19] Jules J, Chen W, Feng X, Li YP (2018). C/EBPα transcription factor is regulated by the RANK cytoplasmic (535)IVVY(538) motif and stimulates osteoclastogenesis more strongly than c-Fos. J Biol Chem.

[20] Jules J, Li YP, Chen W (2018). C/EBPα and PU.1 exhibit different responses to RANK signaling for osteoclastogenesis. Bone.

[21] Jules J, Chen W, Feng X, Li YP (2016). CCAAT/Enhancer-binding Protein α (C/EBPα) Is Important for Osteoclast Differentiation and Activity. J Biol Chem.

[22] Sun Z, Tang Y, Zhang Y, Fang Y, Jia J, Zeng W (2021). Joint single-cell multiomic analysis in Wnt3a induced asymmetric stem cell division. Nature Communications.

[23] Zhu Y, Yu J, Rong Y, Wu Y-W, Li Y, Zhang L (2021). Genomewide decoupling of H2AK119ub1 and H3K27me3 in early mouse development. Science Bulletin.

[24] Yasui T, Hirose J, Aburatani H, Tanaka S (2011). Epigenetic regulation of osteoclast differentiation. Annals of the New York Academy of Sciences.

[25] Adamik J, Pulugulla SH, Zhang P, Sun Q, Lontos K, Macar DA (2020). EZH2 Supports Osteoclast Differentiation and Bone Resorption Via Epigenetic and Cytoplasmic Targets. Journal of bone and mineral research: the official journal of the American Society for Bone and Mineral Research.

[26] Kim J, Shin Y, Lee S, Kim M, Punj V, Lu JF (2018). Regulation of Breast Cancer-Induced Osteoclastogenesis by MacroH2A1.2 Involving EZH2-Mediated H3K27me3. Cell Rep.

[27] Feng H, Xing W, Han Y, Sun J, Kong M, Gao B (2020). Tendon-derived cathepsin K-expressing progenitor cells activate Hedgehog signaling to drive heterotopic ossification. J Clin Invest.

[28] Yang W, Wang J, Moore DC, Liang H, Dooner M, Wu Q (2013). Ptpn11 deletion in a novel progenitor causes metachondromatosis by inducing hedgehog signalling. Nature.

[29] Coussens LM, Tinkle CL, Hanahan D, Werb Z (2000). MMP-9 supplied by bone marrow-derived cells contributes to skin carcinogenesis. Cell.

[30] Kilkenny C, Browne WJ, Cuthill IC, Emerson M, Altman DG (2010). Improving bioscience research reporting: the ARRIVE guidelines for reporting animal research. PLoS Biol.

[31] Addison ML, Rissman EF (2012). Sexual dimorphism of growth hormone in the hypothalamus: regulation by estradiol. Endocrinology.

[32] Chen W, Ma J, Zhu G, Jules J, Wu M, McConnell M (2014). Cbfbeta deletion in mice recapitulates cleidocranial dysplasia and reveals multiple functions of Cbfbeta required for skeletal development. Proc Natl Acad Sci U S A.

[33] Wu M, Li C, Zhu G, Wang Y, Jules J, Lu Y (2014). Deletion of core-binding factor beta (Cbfbeta) in mesenchymal progenitor cells provides new insights into Cbfbeta/Runxs complex function in cartilage and bone development. Bone.

[34] Gao B, Chen W, Hao L, Zhu G, Feng S, Ci H (2013). Inhibiting periapical lesions through AAV-RNAi silencing of cathepsin K. Journal of dental research.

[35] Ma J, Chen W, Zhang L, Tucker B, Zhu G, Sasaki H (2012). RNAi mediated silencing of Atp6i prevents both periapical bone erosion and inflammation in the mouse model of endodontic disease. Infection and immunity.

[36] Sina C, Lipinski S, Gavrilova O, Aden K, Rehman A, Till A (2013). Extracellular cathepsin K exerts antimicrobial activity and is protective against chronic intestinal inflammation in mice. Gut.

[37] Tang CY, Wu M, Zhao D, Edwards D, McVicar A, Luo Y (2021). Runx1 is a central regulator of osteogenesis for bone homeostasis by orchestrating BMP and WNT signaling pathways. PLoS Genet.

[38] Wu M, Chen W, Lu Y, Zhu G, Hao L, Li YP (2017). Galpha13 negatively controls osteoclastogenesis through inhibition of the Akt-GSK3beta-NFATc1 signalling pathway. Nat Commun.

[39] Buenrostro JD, Wu B, Chang HY, Greenleaf WJ (2015). ATAC-seq: A Method for Assaying Chromatin Accessibility Genome-Wide. Curr Protoc Mol Biol.

[40] Zhang Y, Liu T, Meyer CA, Eeckhoute J, Johnson DS, Bernstein BE (2008). Model-based analysis of ChIP-Seq (MACS). Genome Biol.

[41] Mi H, Ebert D, Muruganujan A, Mills C, Albou LP, Mushayamaha T (2021). PANTHER version 16: a revised family classification, tree-based classification tool, enhancer regions and extensive API. Nucleic Acids Res.

[42] Bhuiyan D, Jablonsky MJ, Kolesov I, Middleton J, Wick TM, Tannenbaum R (2015). Novel synthesis and characterization of a collagen-based biopolymer initiated by hydroxyapatite nanoparticles. Acta Biomaterialia.

[43] Zhang Y, Wang H, Zhu G, Qian A, Chen W (2020). F2r negatively regulates osteoclastogenesis through inhibiting the Akt and NFκB signaling pathways. Int J Biol Sci.

[44] Tang CY, Chen W, Luo Y, Wu J, Zhang Y, McVicar A (2020). Runx1 up-regulates chondrocyte to osteoblast lineage commitment and promotes bone formation by enhancing both chondrogenesis and osteogenesis. The Biochemical journal.

[45] Tang C-Y, Wu M, Zhao D, Edwards D, McVicar A, Luo Y (2021). Runx1 is a central regulator of osteogenesis for bone homeostasis by orchestrating BMP and WNT signaling pathways. PLoS genetics.

[46] Tang J, Xie J, Chen W, Tang C, Wu J, Wang Y (2020). Runt-Related Transcription Factor 1 is required for murine osteoblast differentiation and bone formation. J Biol Chem.

[47] Pennica D, Swanson TA, Welsh JW, Roy MA, Lawrence DA, Lee J (1998). WISP genes are members of the connective tissue growth factor family that are up-regulated in wnt-1-transformed cells and aberrantly expressed in human colon tumors. Proc Natl Acad Sci U S A.

[48] Wang M, Sampson ER, Jin H, Li J, Ke QH, Im H-J (2013). MMP13 is a critical target gene during the progression of osteoarthritis. Arthritis Research & Therapy.

[49] Kimbrough A, Houpt TA Nuclear localization of TORC1 and cellular co-localization of TORC1 and c-Fos in the visceral neuraxis after systemic LiCl injection. bioRxiv. 2018: 259812.

[50] Sugatani T, Hruska KA (2005). Akt1/Akt2 and mammalian target of rapamycin/Bim play critical roles in osteoclast differentiation and survival, respectively, whereas Akt is dispensable for cell survival in isolated osteoclast precursors. J Biol Chem.

[51] Maeda K, Kobayashi Y, Udagawa N, Uehara S, Ishihara A, Mizoguchi T (2012). Wnt5a-Ror2 signaling between osteoblast-lineage cells and osteoclast precursors enhances osteoclastogenesis. Nature Medicine.

[52] Duncan EM, Muratore-Schroeder TL, Cook RG, Garcia BA, Shabanowitz J, Hunt DF (2008). Cathepsin L proteolytically processes histone H3 during mouse embryonic stem cell differentiation. Cell.

[53] Duarte LF, Young AR, Wang Z, Wu HA, Panda T, Kou Y (2014). Histone H3.3 and its proteolytically processed form drive a cellular senescence programme. Nat Commun.

[54] Kimura H (2013). Histone modifications for human epigenome analysis. Journal of Human Genetics.

