# Knockout and Double Knockout of Cathepsin K and Mmp9 reveals a novel function of Cathepsin K as a regulator of osteoclast gene expression and bone homeostasis: Erratum

**DOI:** 10.7150/ijbs.142877

**Published:** 2026-10-12

**Authors:** Guochun Zhu, Wei Chen, Chen-Yi Tang, Abigail McVicar, Diep Edwards, Jinwen Wang, Matthew McConnell, Shuying Yang, Yang Li, Zhijie Chang, Yi-Ping Li

**Affiliations:** 1 State Key Laboratory of Membrane Biology, School of Medicine, Center for Synthetic and Systems Biology, Tsinghua University, 100084 Beijing, China; 2 Division in Cellular and Molecular Medicine, Department of Pathology and Laboratory Medicine, Tulane University School of Medicine, Tulane University, New Orleans, Louisiana, 70112, USA; 3 Department of Pathology, University of Alabama at Birmingham School of Medicine, Birmingham, Alabama 35294-2182, USA; 4 Department of Basic & Translational Sciences, School of Dental Medicine, University of Pennsylvania, Philadelphia, PA, USA

The authors regret that an error occurred in Figure 2b. During the final assembly of the figure, we inadvertently selected an incorrect high-magnification ALP image for the WT group instead of the intended image prepared for this manuscript. The corrected figure is provided below, and all authors have agreed to this correction. This correction does not affect the original scientific findings or the conclusions of the study. We sincerely apologize for any inconvenience this error may have caused.

## Figures and Tables

**Figure 2:**
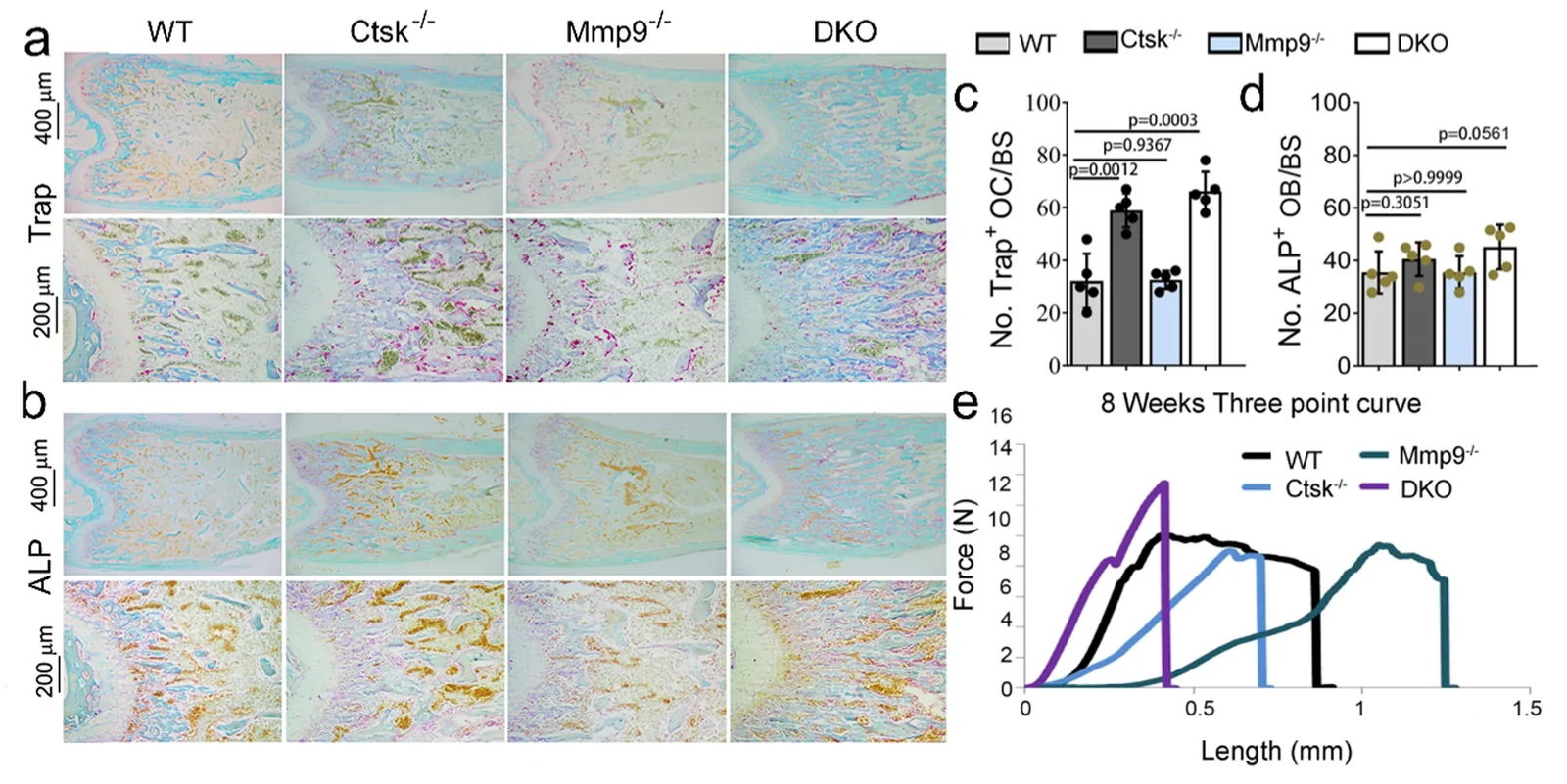
Correct image.

