# Supplementary material for: Knockout and Double Knockout of Cathepsin K and Mmp9 reveals a novel function of Cathepsin K as a regulator of osteoclast gene expression and bone homeostasis
Source: Int J Biol Sci. 2022 Aug 29;18(14):5522–38. doi: 10.7150/ijbs.72211 (PMC9461675; doi:10.7150/ijbs.72211)
Supplement: Supplementary file 1 — Supplementary figures and table. [file ijbsv18p5522s1.pdf]

Knockout and Double Knockout of Cathepsin K and Mmp9 reveals a novel function of Cathepsin K as a regulator of osteoclast gene expression and bone homeostasis

Guochun Zhu[1, 3], Wei Chen[2,3], Chen-Yi Tang[3], Abigail McVicar[2], Diep Edwards[2], Jinwen Wang[3], Matthew McConnell[2], Shuying Yang[4], Yang Li[4], Zhijie Chang[1, *], and Yi-Ping Li[2, 3,*]

Supplemental Material

Primers list for qPCR

gene symbol	Forward primers (5′-3′)	Reverse primers (5′-3′)
c-Fos	CGGGTTTCAACGCCGACTA	TTGGCACTAGAGACGGACAGA
Hprt	GGTGGAGATGATCTCTCAACTTTAA	AGGAAAGCAAAGTCTGCATTGTT
NFATC1	TGCCTTTTGCGAGCAGTATCT	CAGGCAAGGATGGGCTCATAT
Pu.1	CCCCACACCGGCCTCAGTCACCAG	CCCCCACGGCCCAGCACCTC
Cebpα	CAAGAACAGCAACGAGTACCG	GTCACTGGTCAACTCCAGCAC
ATP6i	CACAGGGTCTGCTTACAACTG	CGTCTACCACGAAGCGTCTC

Supplemental Figures and Figure Legends

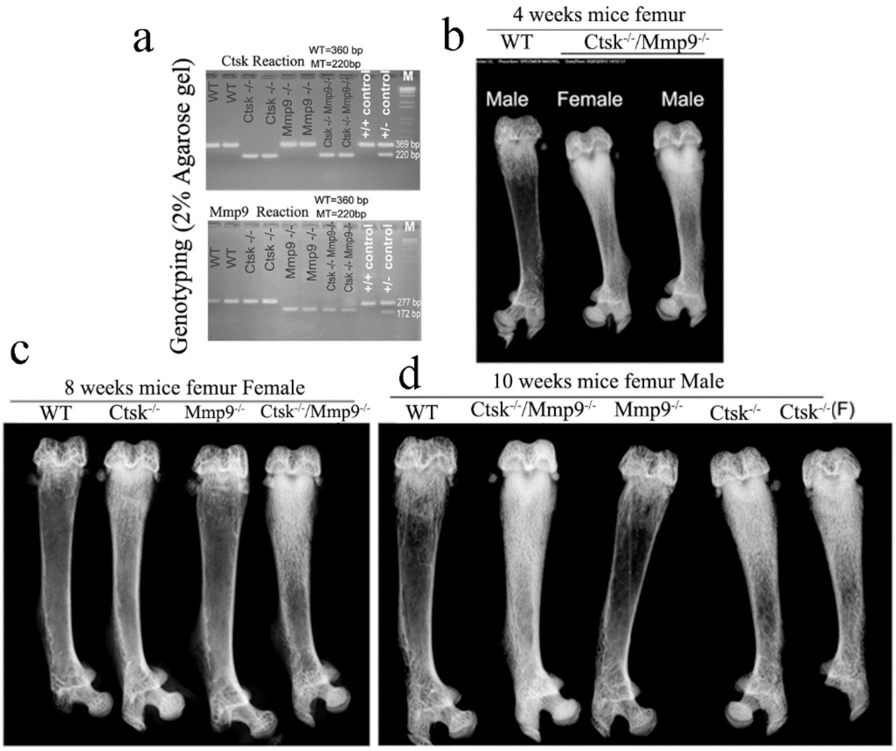

Supplemental Figure 1. Double deletion of *Mmp9* and *Ctsk* causes increased bone mass and severe osteopetrosis in mice. (a) PCR was used to determine the genotypes of the mice. **(b-d)** X-ray analysis of **(b)** 4-week old **(c)** 8-week-old, and **(d)** 10-week-old mouse femurs. Data showed representative images, n=10.

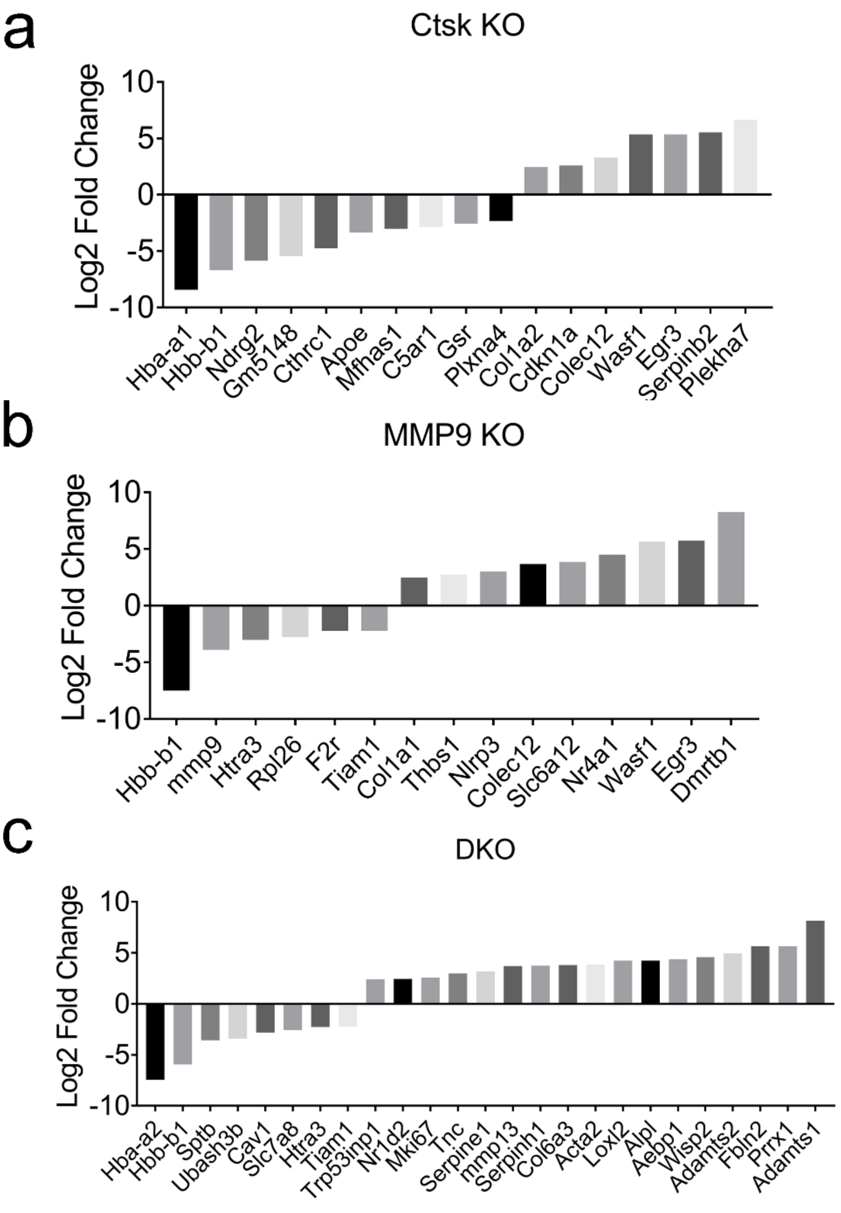

Supplemental Figure 2. RNA-seq analysis of *Ctsk* and *Mmp9* modulated genes. Quantification data from volcano plot illustrating differentially regulated gene expression from RNA-seq analysis between the©ntrol **(c)** *Ctsk*$^{-/-}$, **(b)** *Mmp9*$^{-/-}$ and **(c)** *Ctsk*$^{-/-}$/*Mmp9*$^{-/-}$ osteoclasts from Figure 4a. Values are presented as the log2 of tag counts.

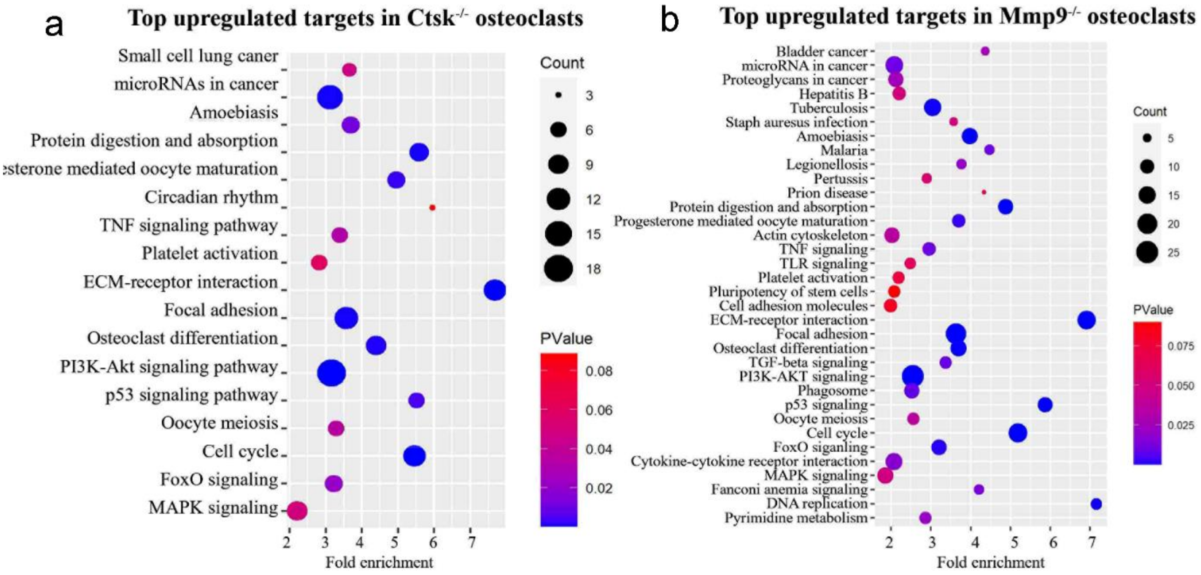

Supplemental Figure 3. RNA-seq analysis of *Ctsk* and *Mmp9* modulated genes in osteoclast signaling. Top upregulated signaling pathways in **(a)** *Ctsk*$^{-/-}$ and **(b)** *Mmp9*$^{-/-}$ osteoclasts, respectively, as analyzed by KEGG database.

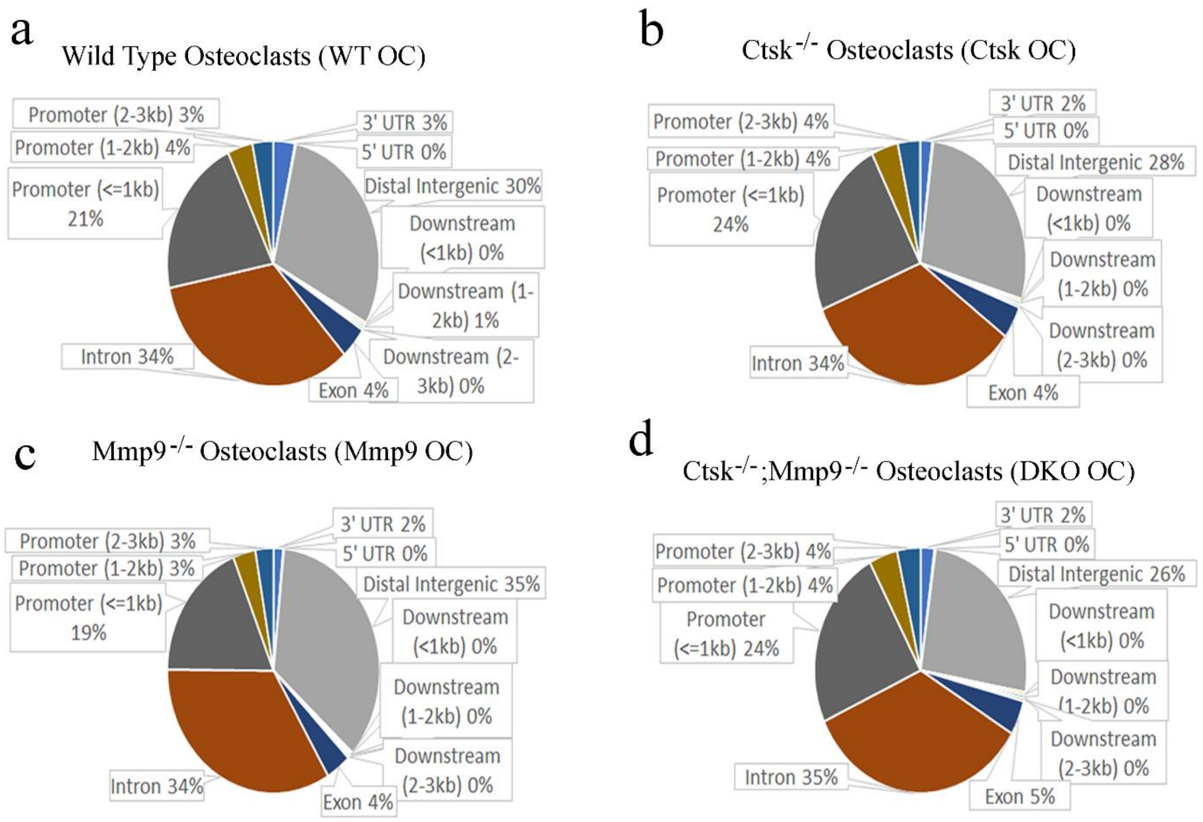

Supplemental Figure 4. ATAC-Seq Profiling of WT, *Ctsk*$^{-/-}$, *Mmp9*$^{-/-}$, and DKO osteoclasts. (a-d) Distribution of ATAC-seq THSs relative to genomic features in **(a)** WT, **(b)** *Ctsk*$^{-/-}$, **(c)** *Mmp9*$^{-/-}$, and **(d)** *Ctsk*$^{-/-}$*;Mmp9*$^{-/-}$ osteoclasts.
